# Microleakage of CEM cement in two different media

**Published:** 2009-07-06

**Authors:** Zahra Ghorbani, Sanam Kheirieh, Bahareh Shadman, Mohammad Jafar Eghbal, Saeed Asgary

**Affiliations:** 1 *Dental Research Center, Dental School, Shahid Beheshti University of Medical Sciences, Tehran, Iran*; 2 *Researcher, Iranian Center for Endodontic Research, Shahid Beheshti University of Medical Sciences, Tehran, Iran*; 3 *Researcher, Dental Research Center, Shahid Beheshti University of Medical Sciences, Tehran, Iran*; 4 *Department of Endodontics, Iranian Center for Endodontic Research, Dental Research Center, Dental School, Shahid Beheshti University of Medical Sciences, Tehran, Iran*

**Keywords:** CEM cement, Distilled water, Fluid filtration, Microleakage, NEC, PBS, Root-end filling

## Abstract

**INTRODUCTION:** Sealing ability of root-end filling materials is of great importance. It can be investigated by measuring microleakage. The purpose of this *in vitro* study was to evaluate microleakage of calcium enriched mixture (CEM) cement in two different media including phosphate buffer solution (PBS) and distilled water.

**MATERIALS AND METHODS:** Twenty single-rooted human teeth were selected. All teeth were root-end filled with CEM cement. Samples were divided into two groups of 10 each and were placed in PBS or distilled water. The microleakage was measured after 12 and 24 h, 14 and 30 days with Fluid Filtration device. Data were statistically analyzed by repeated measures test.

**RESULTS:** Sealing ability of CEM cement was significantly superior in PBS compared to distilled water (P<0.05). This study also showed that time had no significant effect on the sealing ability of CEM cement.

**CONCLUSION:** Media can significantly affect the microleakage of CEM cement. PBS can provide more phosphorous ions for hydroxyapatite formation of CEM cement; therefore, CEM cement can seal more effectively with PBS.

## INTRODUCTION

Microleakage is a well established indicator that assesses sealing ability of root-end filling materials. The measurement of this index is principally based on the penetration of trace agents through the filled canal; these agents include radioisotopes, dyes, and bacteria and their endotoxin (1-3).

Different methods may be used to measure microleakage, some are more reproducible such as fluid filtration and dye extraction techniques (4-7) when compared to scanning electron microscope (SEM) (8) and capillary-flow porometry (9).

One of the root-end filling materials which have been recently introduced is Calcium Enriched Mixture (CEM) cement. It is predominantly composed of calcium compounds (10). Different studies have been performed in order to explore the properties of this material including physical and chemical properties (10) and also composition and surface characteristics (11). Some characteristics have been demonstrated for CEM cement *e.g.* good handling characteristics. In comparison to MTA, CEM cement has a shorter setting time and also significantly superior results in film thickness and flow (10). In addition to the mentioned properties, it has been shown that this material provides acceptable seal; similar to MTA and superior than IRM (12). Asgary *et al.*’s findings also showed that CEM cement has less but not significant microleakage when compared with different types of MTA (13).

Storage media and duration considerably influence the characteristics of dental materials (14-16). Root-end filling materials are in direct contact to blood coagulation products and serum; therefore, using PBS solution *in-vitro *may simulate the *in vivo* condition.

The aim of this *in vitro *study was to investigate the effect of two storage media including PBS and distilled water on the microleakage of CEM cement.

## MATERIALS AND METHODS

The Ethics Committee of Dental Research Center, Shahid Beheshti Medical University, Tehran, Iran approved this study.

Twenty single-rooted human extracted teeth with close apices were included in this experiment. The exclusion criteria were as follows: severe caries, large coronal restorations, root caries, root fractures, dilacerations, deep depressions on root surfaces, two-canalled teeth and those with apical foramen larger than size #35. For the purposes of cross infection control and eliminating soft tissue and periodontal remnants, all teeth were stored in normal saline and were then placed in 5.25% NaOCl solution for 6 hours.

The crowns were resected at standard root length level (10-13 mm). Root canal preparation was performed by crown-down using Gates-Glidden bur size #4-1. The working length was determined and the canals were instrumented with K-files up to size #35. Preparation was carried out using step-back technique by subtracting 0.5 mm length for larger files up to #60. Adequate irrigation was performed during preparation and instrumentation using 2.6% NaOCl.

After resecting apical 3mm end of teeth with tapered diamond bur perpendicular to long axis of the root, we polished root-ends using tapered soft polishing bur. Three-mm depth preparations were made at root ends using ultrasonic power unit (miniPiezon, EMS, Nyon, Switzerland) with ultrasonic retrotips (DT-043, EMS, Nyon, Switzerland) and constant water spray. Samples with detectable cracks were excluded by SEM assessment with ×10 magnifications. Teeth were divided into two experimental groups of ten teeth each. A large file was placed 3 mm short of the apex to provide a stop and prevent coronal penetration of root-end material. The root canal was root-end filled with CEM cement using the largest sized plugger without contacting the root canal walls. Using moist cotton, the excess materials were removed. Root-end filled teeth were randomly divided into two experimental groups (n=10) one of which was stored in 20mL of PBS and the other was stored in 20mL of distilled water. Then the teeth were kept with up-warded apex until the measurement time.

***Fluid Filtration device***

A horizontal capillary with 0.7 mm diameter filled with distilled water and connected it to a vial containing 15 mL distilled water. Using an insulin syringe, water was sucked back into the open end of the glass capillary and an air bubble was created. The roots were inserted into a softened silicone tube, 3 mm in diameter, and placed with cyanoacrylate glue on the outer surface of the tube, avoiding contamination of the apex with glue. The silicone tube was filled with distilled water and connected to the horizontal capillary containing a small air bubble. An observer measured the fluid movement by looking at the bubble vertically after the tooth-device connection. Microleakage was reported as the bubble movement in mm 12 h, 24 h, 14 d and 30 d after root filling.

Using SPSS Version 15 software, repeated measures test was applied to assess the role of time, root filling material and media on the leakage. P<0.05 was assumed significant.

## RESULTS

Descriptive statistics of studied groups are presented in Table 1.

Microleakage assessment of the experimental groups after 12 h, 24 h, 14 d and 30 d showed no significant effect of time on the sealing ability of CEM cement.

Repeated measurement analysis showed that the microleakage of this material was significantly greater in PBS than distilled water (P<0.05).

**Figure 1:**
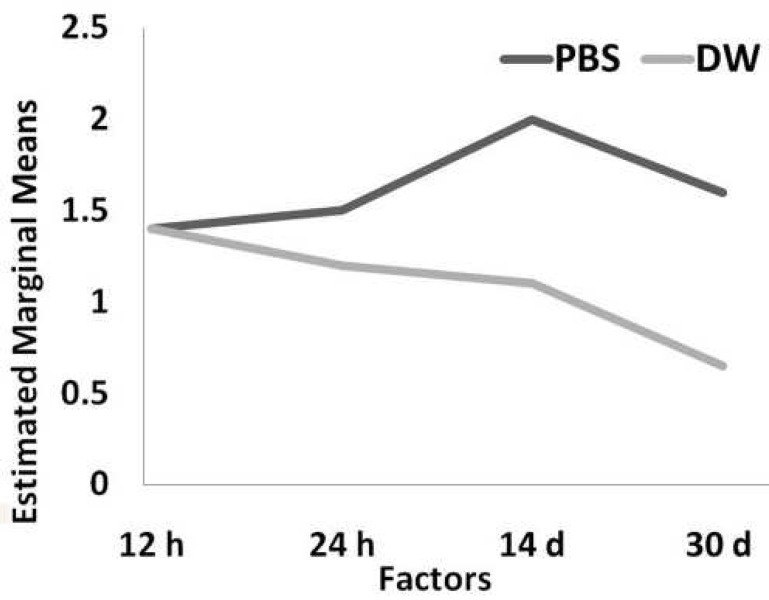
Microleakage of CEM cement in two experimented media

Figure 1 illustrates microleakage of CEM cement in two different studied media.

**Table 1:** Number and mean (standard deviation) of CEM cemet microleakage in two storage media

	12 h	24 h	14 d	30 d
**PBS**^a^	1.4(0.87)	1.5(0.74)	2.0(1.20)	1.6(1.10)
**DW**^b^	1.4(1.09)	1.2(1.06)	1.1(0.74)	0.65(0.88)

^a^ *Phosphate buffer solution,* ^b^ *Distilled water*

## DISCUSSION

Studies on CEM cement showed that this material is dominantly composed of calcium compounds (10) and it is able to release phosphorous and calcium ions which promotes the alkalinity and also lead to mineralization process. Hydroxyapatite formation is demonstrated to be resulted via reaction between calcium and phosphorous ions release (17). It has been shown that CEM cement is able to form hydroxyapatite with endogenous and exogenous sources of phosphorous (17). Sealing ability of CEM cement has been investigated through dye penetration; it has been shown that this root-end filling material provides similar sealing properties to commercial types of MTA (13); this study can be an appropriate complementary investigation as it provides a comparison between sealing ability of CEM cement in two different media.

This study showed that time does not affect the sealing ability of CEM cement; *i.e.* this material reached optimal sealing after 12 h and did not significantly change over time. MTA demonstrated the same results only after 24 h; this may be due to shorter setting time of CEM cement.

Present study shows that microleakage of CEM cement in PBS is significantly less than distilled water. This can be due to the promotion of mineralization and hydroxyapatite formation that CEM cement induces by the presence of exogenous sources of phosphorous which are provided by PBS. Another study demonstrated secondary seal provided by CEM cement via hydroxyapatite formation over this new material (17).

Fluid transport method was used in this study because of several advantages over common dye penetration method some of which are that samples are not destroyed, it permits the evaluation of the sealing efficiency over time, and also quantitative measurement of microleakage in small ranges (5). This system has some disadvantages; for example, it is time-consuming to set-up and measure, is technique-sensitive and, if not automatized, the reliability of the measurements depends on the precision of the naked eye. Additionally, no exact data on the size of the pores can be obtained (18). The measurements in this study were performed by blinded observer in order to avoid inter-examiner bias.

## CONCLUSION

According to the present study, storage media affects the sealing ability of CEM cement. This material is able to provide a greater seal in the presence of PBS, a phosphorous rich media. For additional similar simulation of the clinical condition, we can suggest the use of blood serum in future studies.
